# Modeling of Urinary Microbiota Associated With Cystitis

**DOI:** 10.3389/fcimb.2021.643638

**Published:** 2021-03-16

**Authors:** Marina Ceprnja, Damir Oros, Ena Melvan, Ema Svetlicic, Jasenka Skrlin, Karmela Barisic, Lucija Starcevic, Jurica Zucko, Antonio Starcevic

**Affiliations:** ^1^ Biochemical Laboratory, Special Hospital Agram, Polyclinic Zagreb, Zagreb, Croatia; ^2^ Department of Medical Biochemistry and Hematology, Faculty of Pharmacy and Biochemistry, Zagreb University, Zagreb, Croatia; ^3^ Laboratory for Bioinformatics, Faculty of Food Technology and Biotechnology, Zagreb University, Zagreb, Croatia; ^4^ Department of Biological Science, Faculty of Science, Macquarie University, Sydney, NSW, Australia; ^5^ Department for Clinical Microbiology and Hospital Infection, University Hospital Dubrava, Zagreb, Croatia

**Keywords:** urinary tract infection, microbiome, 16S rRNA sequencing, microbial interaction modeling, antibiotics, therapy duration

## Abstract

A decade ago, when the Human Microbiome Project was starting, urinary tract (UT) was not included because the bladder and urine were considered to be sterile. Today, we are presented with evidence that healthy UT possesses native microbiota and any major event disrupting its “equilibrium” can impact the host also. This dysbiosis often leads to cystitis symptoms, which is the most frequent lower UT complaint, especially among women. Cystitis is one of the most common causes of antimicrobial drugs prescriptions in primary and secondary care and an important contributor to the problem of antimicrobial resistance. Despite this fact, we still have trouble distinguishing whether the primary cause of majority of cystitis cases is a single pathogen overgrowth, or a systemic disorder affecting entire UT microbiota. There are relatively few studies monitoring changes and dynamics of UT microbiota in cystitis patients, making this field of research still an unknown. In this study variations to the UT microbiota of cystitis patients were identified and microbial dynamics has been modeled. The microbial genetic profile of urine samples from 28 patients was analyzed by 16S rDNA Illumina sequencing and bioinformatics analysis. One patient with bacterial cystitis symptoms was prescribed therapy based on national guideline recommendations on antibacterial treatment of urinary tract infections (UTI) and UT microbiota change was monitored by 16S rDNA sequencing on 24 h basis during the entire therapy duration. The results of sequencing implied that a particular class of bacteria is associated with majority of cystitis cases in this study. The contributing role of this class of bacteria – *Gammaproteobacteria*, was further predicted by generalized Lotka-Volterra modeling (gLVM). Longitudinal microbiota insight obtained from a single patient under prescribed antimicrobial therapy revealed rapid and extensive changes in microbial composition and emphasized the need for current guidelines revision in regards to therapy duration. Models based on gLVM indicated protective role of two taxonomic classes of bacteria, *Actinobacteria* and *Bacteroidia* class, which appear to actively suppress pathogen overgrowth.

## Introduction

Human microbiome is a fairly new concept, fueled by the advances in sequencing technology which allowed identification of microorganisms directly from the environment (Oulas et al., 2015). Combined with advances in computational tools and databases it allowed for identification of microorganisms living in and on our body - microbiota, as well as reading out the collective genome encoded in those microorganisms – microbiome (Qin et al., 2010). UT is one of the last body niches discovered to be inhabited by microorganisms. Microbiota inhabiting a healthy UT is dominated by slow-growing species, which require complex nutrients for growth and are often missed during standard urine culture (SUC) procedure (Brubaker and Wolfe, 2016). Reason for that is the long prevailing dogma about sterility of the UT, recently overturned thanks to advances in sequencing methods and enhanced urine culture methods (Whiteside et al., 2015; Thomas-White et al., 2016). Discovery of urinary microbiome (Nelson et al., 2010; Wolfe et al., 2012; Lewis et al., 2013; Hilt et al., 2014; Brubaker and Wolfe, 2015) shifts our perception about treating UTI as it acknowledges normal presence of microorganisms not causing inflammation symptoms. Whether composition of “normal” urinary microbiota has a protective, detrimental or neutral role in it is still in focus of research. UTI being one of the most commonly diagnosed infections in the world, especially among women present great burden to health systems and to quality of life of affected individuals, especially in case of recurring infections. Urinary microbiota, unlike microbiota at other body niches, has low-biomass and fairly low species richness, usually dominated by one or two species. Some studies are suggesting that majority of organisms identified in healthy humans are common to men and women and that male urinary microbiota is just a subset of female one (Gottschick et al., 2017). Others suggest male and female urinary microbiota can be distinguished based on abundance of specific genera, with higher abundance of *Lactobacillus* and *Prevotella* genera being characteristic for female and higher abundance of genus *Corynebacterium* for healthy male urinary microbiota (Fouts et al., 2012). By sequencing and culture techniques, most commonly found microbes in healthy females at a genus level are *Lactobacillus*, followed by *Gardnerella, Corynebacterium, Streptococcus*, and *Staphylococcus* species (Wolfe et al., 2012; Mueller et al., 2017; Neugent et al., 2020). Due to the fact that male and female lower UT are structured differently, it is challenging to distinguish from healthy male and female urinary microbiota. Current SUC results have shown urinary microbiota during an UTI is dominated by standard urinary pathogens such as uropathogenic *Escherichia coli*, *Klebsiella, Pseudomonas*, and *Enterobacter* species, but have also identified novel bacteria associated with infection such as *Acidovorax*, *Rhodanobacter*, and *Oligella* species (Flores-Mireles et al., 2015; Moustafa et al., 2018). However, innate limitations of SUC, which relate to culturing conditions and differences in growth media, differences in interpretation dependent on clinical condition and time to completion of findings have to be considered when interpreting these studies results. UTI-related urinary microbiota’s metagenome showed a number of antimicrobial resistance genes present, questioning efficacy and wisdom of prescribing antibiotic treatment without determining antibiotic sensitivity (Hasman et al., 2014; Mulder et al., 2019). Applying oral antibiotics for UTI treatment has led to decrease in the diversity of urinary microbiota and impacted “equilibrium” of microorganisms present, with unexpected increase in abundance of *Lactobacillus* species (Siddiqui et al., 2012). However, there are a few studies exploring the link between urinary microbiota, UTIs and antibiotic use (Gottschick et al., 2017; Mulder et al., 2019). Excessive and irrational use of antibiotics results in over-treatment of cystitis and promotes growth of antibiotic resistant strains (Frost et al., 2019; Malik and Bhattacharyya, 2019). In order to change this negative trend, two major therapy parameters have to be reassessed. Although the first parameter, antimicrobial susceptibility is satisfactory, being performed routinely, the second one - therapy duration is much more difficult to assess. Despite good evidence that shorter duration of antibiotics therapy have led to less adverse effects (Jancel and Dudas, 2002; Milo et al., 2005), optimal duration is still largely unrecognized by medical community. Unfortunately, this leads to rather arbitrary duration of treatment (“magic” numbers such as 7, 10, and 14 days), being prescribed to patients in practice (Pezzani et al., 2020). The main reason for this is mainly historical; due to the fact that antibiotics research is neglected by modern pharma (Harbarth et al., 2015) we rely on outdated studies. Beside this issue, a rather slow take-up of new technologies coming from genomics and proteomics in medical practice is causing a gap between cutting edge research in fields such as precision medicine on one side and primary care on the other (Ho et al., 2020). The fact that highly interdisciplinary teams are conducting research while primary care physicians usually work alone is probably the main reason why this gap will not be closing any time soon.

In this work, we present the results of 16S rRNA sequencing of urinary microbiota from 28 patients with suspected cystitis. Moreover, we have investigated a daily change in urine microbiota content in a patient receiving prescribed antibiotic treatment based on current national guidelines. Illumina sequencing and bioinformatics estimated relative abundances of taxa comprising patients urinary microbiota were used to build generalized Lotka-Volterra models (gLVM), which indicated a common taxonomic denominator in all analyzed urine samples. Furthermore, gLVM suggested that interactions between major taxonomy classes or UT microbiota modeled according to simple predator-prey dynamics indicate protective role of bacteria belonging to *Actinobacteria* and *Bacilli* classes in case of bacterial cystitis associated with *Gammaproteobacteria* class pathogen. Monitoring of one patient microbiota for the entire duration of prescribed antimicrobial therapy on 24 h basis, suggested that current national guidelines on antimicrobial treatment and prophylaxis of urinary tract infections should be revised when it comes to therapy duration.

## Materials and Methods

### Sample Collection and Urinary Culture

The Ethics Committee of University Hospital Dubrava approved the study protocol. Thirty-five urine samples were collected from 28 patients in Department for Clinical Microbiology and Hospital Infections of the University Hospital Dubrava in Zagreb, Croatia. Samples were collected in the period from January to December 2016. All patients were volunteers and gave the informative consent to donate their urine samples for research purposes. There were 16 females and 12 male participants and the average age was 66. In addition to urine samples collected by clean catch method where each patient provided first voided morning urine, six hospitalized catheter patients were included in the study; patient demographics can be seen in **Supplementary Figure 1**.

The initial cystitis diagnosis was based on SUC test, as described previously (Oros et al., 2020). Inclusion criteria was bacterial infection with >10^5^ CFU/ml before introducing any antibiotic therapy. No antibiotic therapy was started until SUC result returned >10^5^ CFU/ml result. Longitudinal study of antibiotic influence on urobiome was monitored on 38-year-old female patient with acute cystitis symptoms. Urine samples were collected from the same female patient in form of first-void specimen for 8 consecutive days during which this female patient was taking 1 g daily monodose of Cephalexin (beta-lactam antibiotic which belongs to class of first-generation cephalosporins) oral therapy, as prescribed. From the eight samples taken, first sample was collected after antimicrobial susceptibility test and before starting the antibiotic therapy, and the other 7 were taken successively after starting antibiotic therapy in 24 h interval. Routine hospital microbiology SUC was repeated on three samples (1^st^, 4^th^, and the last one). All urine samples were stored at minus 20°C prior to further processing.

### Sequencing of 16S rRNA Genes and Subsequent Bioinformatics Analysis

Frozen samples were thawed at room temperature and homogenized volume of 1.5 ml urine sample was centrifuged at 10,000 × g for 5 min at 4°C, and supernatant was removed. DNA was isolated from the urine pellet using a Maxwell 16 Cell DNA Purification Kit on the Maxwell 16 research instrument (Promega, Madison), according to the manufacturer’s protocol. DNA concentrations were measured using a BioSpec-nano spectrophotometer (Shimadzu Biotech), and samples were stored at −80°C. Extracted DNA from all 35 urine samples was used to sequence regions V3 and V4 of the gene coding for 16S rRNA using the Illumina MiSeq platform following paired-end sequencing protocol. Quantitative Insights into Microbial Ecology 2 - QIIME2 (Bolyen et al., 2019) was used to perform the analysis of sequenced reads. Raw data, obtained from Illumina’s BaseSpace as fastq files, was demultiplexed and quality filtered using q2-demux plugin and subsequently denoised using DADA2 (Callahan et al., 2016). Taxonomy was assigned to amplicon sequence variants (ASV) using the naive Bayes taxonomy classifier, as implemented in QIIME2, against the Greengenes 13_8 99% OTUs as reference taxonomic sequences (McDonald et al., 2012). A total number of features at the taxonomic level family (Level 5) were 468,817 with an average number of features per sample being 17,363. The microbial diversity and sample completeness were assessed using rarefaction curve of alpha diversity, estimated using the q2-diversity plugin with samples rarefied to 1130 sequences per sample. The rarefaction curve of observed OTU-s dependent on sequencing depth is shown in **Supplementary Figure 2**, showing that all samples except LS2 reached saturation. In urine samples U1-U27 the average number of OTUs at the maximum sequencing depth is 24.7, minimal observed depth being 3 and maximal 107. The average number of OTUs in urine samples where the antibiotic effect was monitored (LS1-LS8) was 54.1. Initial number of OTUs before the first Cephalexin dose was administered was 30 (LS0), while the highest number of OTUs (123) was observed on second day of antibiotic therapy (LS2) and the lowest number of OTUs of 21 was observed on day 7 (LS7). The data can be found in **Supplementary Dataset 1**.

### Learning Directed Microbial Interactions From Cross-Sectional Microbiome Profiling Data Based on the Generalized Lotka-Volterra Model

In order to learn directed microbial interaction from cross-sectional microbiome profiling data BEEM-static R package (Li, 2020a) was utilized. BEEM-Static is based on generalized Lotka-Volterra model (Metz et al., 1996; Hofbauer and Sigmund, 1998) designed for cross-sectional datasets in order to learn and model the directed microbial interactions (Li, 2020a). BEEM-static in an extension of the original BEEM algorithm that enables a precise ecological modeling based on the microbiome sequencing longitudinal data (Li et al., 2019). Working directly with the relative abundances, the experimental measurement of the absolute abundances is unnecessary and therefore suitable for microbiome datasets where the equilibrium status is unknown. The input file for BEEM-static was an OTU table containing the number of sequences that were observed in each taxonomic unit in each sample, provided by QIIME. In this study, 249 different taxonomic units were found on strain level. The model was built on all taxonomy levels and the coefficient of determination, denoted R^2^, was the highest on the class level (Steel et al., 1960; Glantz et al., 2016). Because the coefficient of determination provides a measure of how well observed outcomes are replicated by the model, class level was chosen as a baseline. Therefore, all OTUs were grouped on a class taxonomic level, creating 35 different class taxonomic units. To remove the OTUs not detectable in the majority of samples and reduce the number of OTUs for a model, the original OTU table was filtered to keep only top OTUs based on prevalence (OTU is found in at least half of the samples), which was later transformed into relative abundance. Usually, the default criteria are to keep the OTUs that are found in at least 25% of the overall samples. However, when these default criteria were used, only one more OTU was retained in the model calculation. Due to our restricted number of samples and the fact that BEEM-static has been tested on >4,000 microbiome data points, we decided to use 50% prevalence as a more stringent criteria. In both 25% and 50% prevalence, the same OTUs are still significant and because overall they are the most abundant, any pruning variant in-between these criteria would lead to the same outcome. Microbiomes, which were not in equilibrium states, were detected by BEEM-static and automatically removed from the further analysis. In the pooled dataset originating from a cohort consisting of 28 volunteers, seven classes were found in at least 50% of samples and were kept. All seven were in equilibrium state and were used to build the model. In the second dataset, obtained from single patient receiving an antibiotic therapy for 7 days, out of 11 classes present in at least 50% of samples, seven classes were not in equilibrium and were thus removed. Remaining four classes were used to build the model. The package was obtained from GitHub (Li, 2020b) and installed in RStudio (version 1.3.959) under R-4.0.2 on Windows 10 computer. Both the input data and dedicated R scripts used in this study can be accessed at https://github.com/enmelvan/Dynamics-of-urinary-microbiota-associated-with-cystitis.

## Results

### Study Design

A gender-balanced cohort consisting of 28 volunteers (16 female, 12 male) referred to SUC test for cystitis symptoms was involved in the study after signing the informative consent. The initial diagnosis was based on patient’s symptom description and further confirmed by standard urine culture test. Culture test inclusion criteria were associated with bacterial infection characterized by > 10^5^ colony-forming units per ml (CFU/ml). The average age of patients was 66 years. In addition to urine samples collected by clean catch method, small number of catheterized patients was included in the study. One patient with uncomplicated cystitis symptoms caused by common urinary pathogen was singled out and included in longitudinal study in order to monitor dynamics of UT microbiota under prescribed treatment. Beside SUC tests, all samples were subjected to Illumina 16S rRNA sequencing. The goal of the study was to compare SUC tests with genomics based tests and to assess the link between urinary microbiome and cystitis. The goal of the longitudinal study monitoring urinary microbiome dynamics under prescribed antimicrobial therapy was to investigate the impact of the commonly prescribed antimicrobial therapy on the urinary microbiota and to assess the possibility of recommending optimal duration of the antimicrobial therapy.

### SUC Reveals Infections Caused by Common Uropathogens

Out of total 28 subjects, SUC indicated 19 monobacterial infections related to *Proteus mirabilis* (five patients), *Klebsiella* spp. (four patients), *Enterobacter* spp. (three patients), *Escherichia coli* (three patients), *Enterococcus faecalis* (two patients), and *Pseudomonas aeruginosa* (two patients). Polymicrobial infections were associated with seven patients (samples U1, U4, U6, U7, U19, U24, and U25). Urine culture test results of all samples are displayed in **Supplementary Table 1**.

The influence of antibiotic therapy on urinary microbiome was investigated on a patient whose cystitis symptoms were linked to common bacterial pathogen by SUC displaying > 10^5^ CFU/ml of *K. pneumoniae*. Based on recommendations of clinical practice guidelines in Croatia (Skerk et al., 2009), an antibiogram was used to prescribe standard antibiotic therapy in optimal duration of seven days. Antimicrobial susceptibility test indicated that infection caused by *K. pneumonia* strain was resistant to Ampicillin thus the patient was prescribed Cephalexin (Cefalexin). As part of the study, SUC monitoring was performed on the third and final, seventh day of prescribed antibiotic therapy. On the third day of antibiotic therapy SUC revealed no pathogen and urine was proclaimed sterile. After completing the antibiotic therapy course for the entire 7 days, SUC test indicated *Candida albicans* infection in concentration >10^3^ and <10^5^ CFU/ml.

### Taxonomic Profiling of UT Microbiota

**Figure 1:**
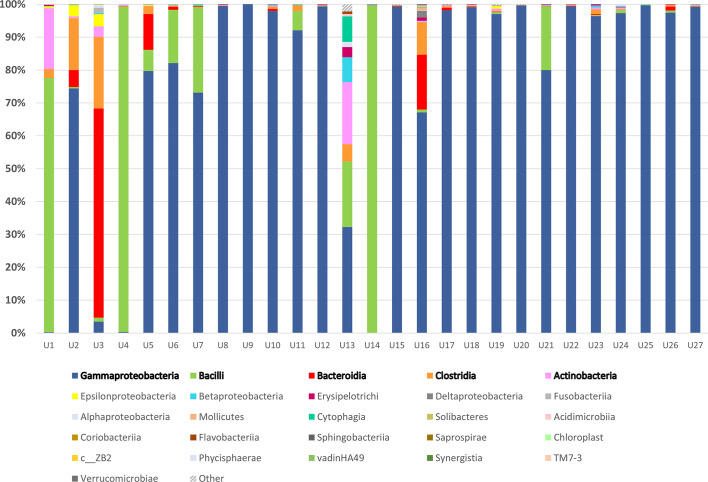
16S rRNA sequencing of urine samples from patients diagnosed with UTI: Taxonomic representation of bacterial classes as identified by sequencing of V3 and V4 region of 16S rRNA gene. The most abundant classes were marked in bold and sequences belonging to group marked as “Other” were not matched to any sequence in Greengenes database.

There were 15 distinct phyla shared by all 28 urine samples collected from all study included patients, out of which *Proteobacteria*, *Firmicutes*, *Bacteroidetes, and Actinobacteria* accounted for more than 99% of detected taxa. Classes *Gammaproteobacteria* and *Bacilli* dominated across all samples (**Figure 1**). Single-family dominance was observed in 19 urine samples in which *Enterococcaceae*, *Enterobacteriaceae*, or *Pseudomonadaceae* contributed with over 90% of total bacterial abundance. The more diverse spectrum of bacteria was observed in samples U1, U2, U3, U5, U13, and U16 in which more than four bacterial families were identified. 16S rRNA sequencing successfully identified 123 different genera (**Supplementary Dataset 1**), while the species rank was missing in majority of samples due to inherent limitations of 16S rRNA sequencing (Poretsky et al., 2014), therefore the results of 16S rRNA sequencing were compared with SUC at the family taxonomic level. SUC results were largely in accordance with 16S rRNA sequencing, although a discrepancy between two methods was observed in samples U1, U3, and U13 (**Supplementary Table 1**).

### Dynamics of Urinary Microbiota During Cystitis

**Figure 2:**
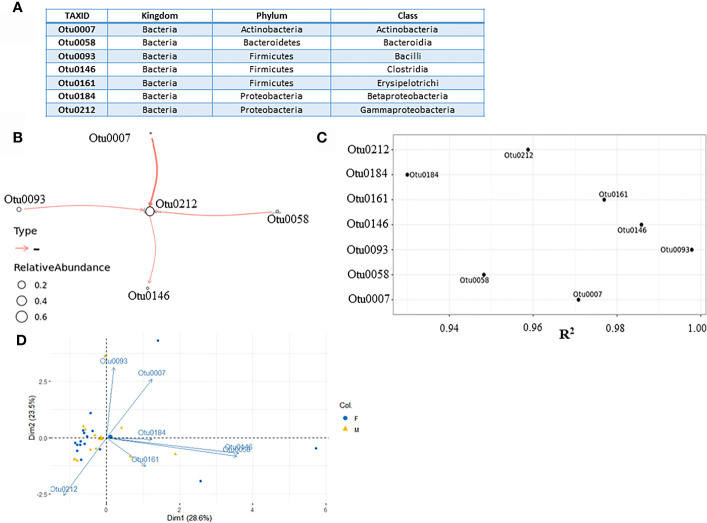
Microbial interaction network based on microbiome profiling of 28 urine samples from patients displaying cystitis symptoms. **(A)** Seven most abundant classes used for modeling. **(B)** Coefficient of determination R^2^ for each class input. **(C)** Network graph representing nonzero interaction terms in gLVM models learnt individually from urine microbiome profiling using BEEM-static. Graph edges in red represent negative interactions. Edge widths are proportional to the interaction strength, and node sizes are proportional to the log-transformed mean relative abundance of the corresponding class. Nodes are labeled with the class level taxonomic annotations.** (D)** PCA biplot displaying microbiome variation between male and female patients.

We have used BEEM-static (Biomass Estimation and model inference with an Expectation Maximization) to infer a model based on 16S rRNA sequencing data for microbial community dynamics (Li et al., 2019). This expectation-maximization algorithm is based on generalized Lotka-Volterra (gLVM) ecological model that can provide useful insights into microbial interactions and dynamics (Venturelli et al., 2018). Before using BEEM-Static, principal-component analysis (PCA) based on patient gender and most abundant operational taxonomic units (OTUs), was used to assess heterogeneity of urinary microbiome. PCA biplot indicated that male and female samples cannot be clearly separated and therefore we modeled them together and not separately (**Figure 2D**).

The input file for BEEM-Static was an OTU table containing the number of sequences that are observed in each taxonomic unit in each sample. In this study, 249 different taxonomic units were found on taxonomic levels ranging from kingdom to species (**Supplementary Dataset 1**). They were grouped on a class taxonomic level, creating 35 different class taxonomic units. To reduce the number of OTUs in order to use BEEM-Static model, seldom appearing OTUs were removed. The original OTU table was filtered based on the prevalence of OTUs, retaining only those found in > 50% of the samples. In this manner, seven most abundant OTUs (**Figure 2A**) were transformed from counts into relative abundances across all 28 samples, creating an input file for BEEM-static (**Supplementary Dataset 2**). Microbial interaction network based on mapping file for seven most abundant classes is shown below (**Figures 2B, C**). Classes which do not appear in the network, despite being abundant (Otu161 and Otu184) are considered to be neutral by model. Regarding the interactions depicted by the model, the model was generally aligned with PCA biplot made previously, despite the fact that Dimensions 1 and 2 retained about 52% (28.6% + 23.5%) of the total information contained in the data set.

*Gammaproteobacteria* class (Otu0212), which was positioned at the core of the model appears to be a common denominator taxa in all tested urine samples. There was one notable negative interaction between *Actinobacteria* class (Otu0007) negatively impacting *Gammaproteobacteria*, which most of the UT pathogens belong to. This negative interaction was predicted to be stronger than interaction between both *Bacilli* (Otu0093) and *Bacteroidia* (Otu0058) classes and *Gammaproteobacteria* class, which implied that *Actinobacteria* relative abundancy was negatively correlated with the level of *Gammaproteobacteria* in affected individuals. The coefficient of determination (R^2^) is the proportion of the variance in the dependent variable that is predictable from the independent variable(s) (Steel et al., 1960; Glantz et al., 2016). Since in our case, there were no independent variables, we argue that relatively high R^2^ (close to 1) indicated that the variation in the data was well explained by the model. According to R^2^ values (**Figure 2B**), all seven classes which were included, could be described by the model.

### The Urinary Microbiota Under the Influence of Antibiotic Therapy

**Figure 3:**
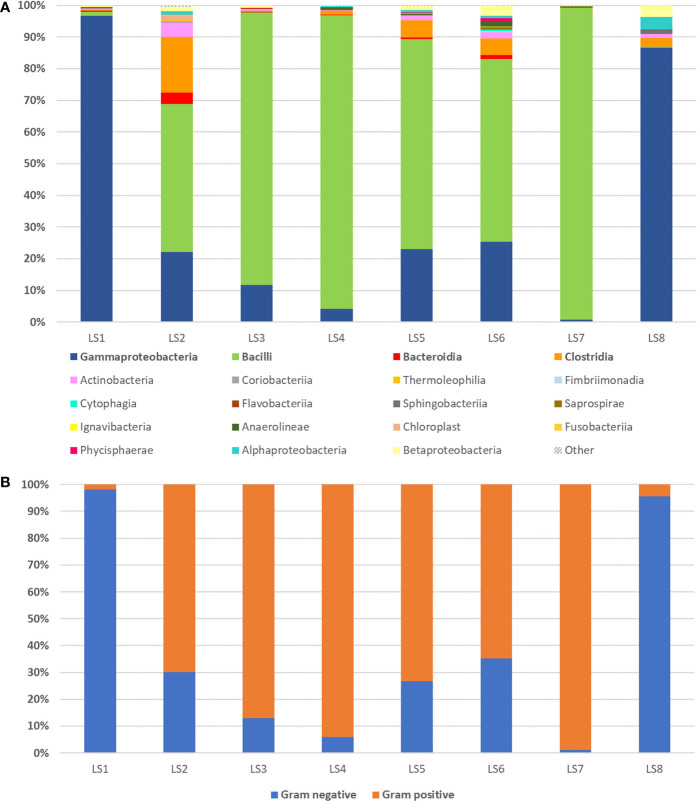
16S rRNA sequencing of urine samples during and 7-day antibiotic therapy observed in single patient with cystitis symptoms caused by *K. pneumoniae* infection determined with standard urinary culture test. **(A)** Eight day period measurements of relative abundance of bacterial classes detected by urine sample 16S rRNA sequencing coming from a single patient receiving antibiotic therapy for 7 days (first measurement taken one day prior therapy). Group “Other” represents organisms, which contributed with less than 1% or could not be assigned. **(B)** Bar chart displaying a change in Gram-positive and Gram-negative bacteria abundance during 7-day antibiotic therapy with Cephalexin monodose, 1 g per day.

The taxonomic distribution of urinary microbiota for the single patient receiving an antibiotic therapy for 7 days is shown in **Figure 3**. Before administration of Cephalexin, Gammaproteobacteria were identified as a predominant class, further confirming previous SUC indicated *Klebsiella pneumoniae* infection. In a sample taken 24 h after taking the first antibiotic monodose, *Enterobacteriaceae*, which constituted over 95% of all bacteria detected 24 h before starting therapy, were reduced to 1.28%. Concurrently, heterogeneity of overall microbiota deepened, accompanied by rise in families *Lactobacillaceae* and *Pseudomonadaceae*. Diversity of microbiota was lower on days 3–4 with over representation of genus *Lactobacillus*, which comprised 85.5% of microbiota on day 3 and 91.9% on day 4, based on QIIME results. While an increase of diversity is shown on days 5 and 6, *Lactobacillus* spp. was relatively most abundant on day 7. On day 8, the last day of 1 g monodosis oral Cephalexin therapy, family *Pseudomonadaceae* had dominated microbiota with relative abundance of 74.61% (**Figure 3A**).

In this period, ratio of Gram-positive to Gram-negative bacteria in urine samples altered dramatically. Gram-negative bacteria relative abundance has been gradually decreasing from day one to day 4, followed by its increase on days 5 and 6 (**Figure 3B**).

### Dynamics of Urinary Microbiota Under Antibiotic Influence

**Figure 4:**
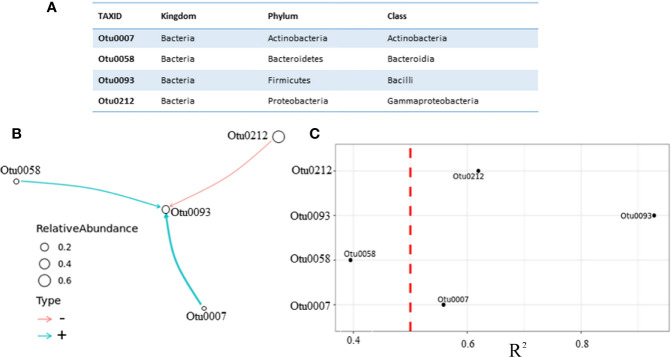
Microbial interaction network based on daily monitoring of urine from a single patient under prescribed antibiotic therapy. **(A)** Four most abundant classes, which were in equilibrium state **(B)** Network graph representing nonzero interaction terms in gLVM models learnt individually from urine microbiome profiling using BEEM-static. Graph edges in red represent negative interactions and blue edges represent positive. Edge widths are proportional to the interaction strength, and node sizes are proportional to the log-transformed mean relative abundance of the corresponding class. Nodes are labeled with the class level taxonomic annotations described in the table. **(C)** Coefficient of determination (R^2^) for each class used by the model, vertical dashed line depicted in red color was set at R^2^ = 0.5, indicating that interaction with Otu0058 (R^2^ = 0.39) should be taken with caution.

In order to model the change of the microbiota during antibiotic therapy on 24 h basis, the original BEEM algorithm for longitudinal data was used. The input data for BEEM (Li et al., 2019) consisted of an OTU table containing the number of sequences that were observed in each taxonomic unit in each sample and metadata containg time measurements (**Supplementary Dataset 3**). Different taxonomic units were grouped on a class taxonomic level, creating 35 different classes. Only the most abundant OTUs (appearing in > 50% of urine samples) were used to construct the model (**Figure 4A**). In this case, four OTUs were normalized from counts into relative abundances across all eight samples, serving as input data for BEEM model. Microbial interaction network based on those OTUs is displayed in **Figure 4B**.

**Figure 5:**
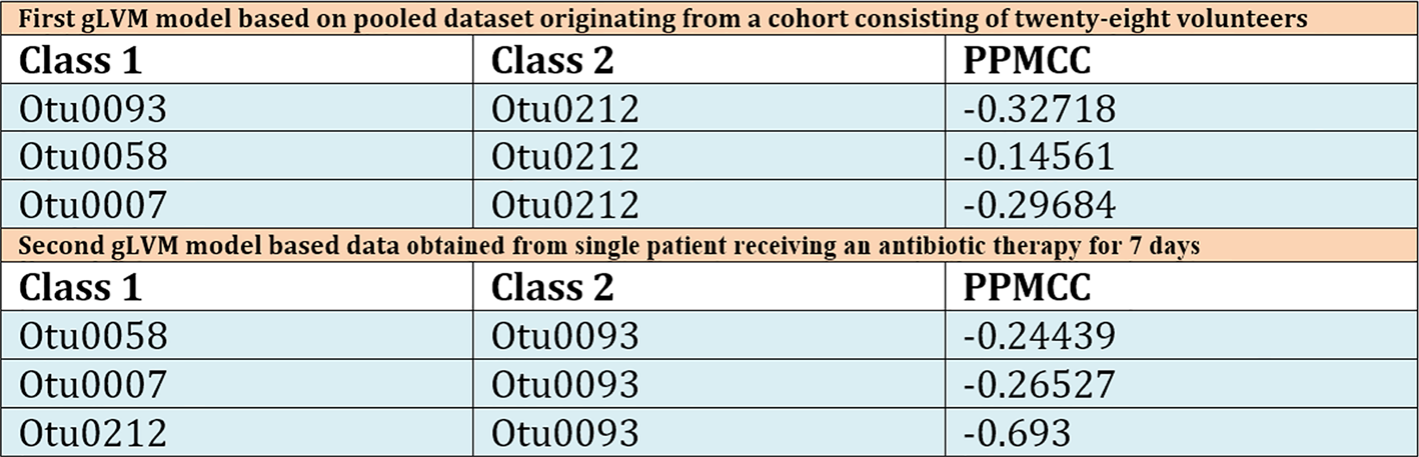
Table displaying calculated Pearson Product Moment correlation coefficients for all model inferred interactions for both pooled patient dataset and single patient dataset. In the table, column marked Class 1 corresponds to model’s interaction network outlier classes, while Class 2 denotes the class centered at the core of model. Third column contains calculated Pearson Product Moment correlation coefficients (PPMCC). This is a measure of linear correlation between two sets of data, and although the results obtained can be correlated with those obtained by the gLVM model based on pooled patient dataset, direct comparison is nontrivial because PPMCC ignores many other types of relationship or correlation.

Unlike in the previous model, which was based on different patients with no antibiotic therapy, this model puts *Bacilli* class (Otu0093) at the core of the model, and this class appears to be negatively impacted by the *Gammaproteobacteria* class (Otu0212), which is the most abundant one. This model revealed one more difference regarding two positive interactions, weaker one between *Bacteroidia* class (Otu0058) and *Bacilli* (Otu0093) and stronger one between *Actinobacteria* (Otu0007) and *Bacilli* (Otu0093) classes. We argue that in this patient receiving antibiotic therapy *Actinobacteria* class appears to be postively correlated with *Bacilli* class *Lactobacillus* genus belongs to, while the *Gammaproteobacteria* class appears to be negatively correlated with *Bacilli*, which favors the hypothesis of *Lactobacillus* protective role. Having said this, the question remains whether the choice of gLVM is really appropriate? Since no method is perfect, BEEM estimations of gLVM parameters are not error free. Therefore, we performed a simple correlation analysis, which included taxa used by our models in order to further asses inferred interactions between them (**Figure 5**).

## Discussion

Overall, our research indicates that major phyla *Proteobacteria*, *Firmicutes*, and *Bacteroidetes*, which were detected in urine samples of subjects with cystitis symptoms, as well as relatively low number of genera per sample, are in accordance with previous research dealing with cystitis patients (Siddiqui et al., 2012). The biological significance of predominance by any specific organism or the lack of a predominant microbe is not yet known. However, our results indicate overabundance of one class of bacteria in the urine of patients with cystitis symptoms— *Gammaproteobacteria*, was the main causative agents of cystitis in our study. Although urinary microbiota is low complexity in comparison with other bodily niches (Karstens et al., 2018), overrepresentation of an OTU belonging to a single class might indicate that OTU “equilibrium” in urine microbiota could be another equally important contributing factor.

In our longitudinal study, we had selected a single female patient. This patient was selected because of clear clinical presentation, having a last documented SUC finding of 10^5^ CFU/ml *Lactobacillus* species and no cystitis symptoms for at least 1 year, based on patient’s recollection and medical exams in this period. Because this patient was pregnant, we were able to establish sufficiently strict monitoring in order to rule out antibiotics usage and unreported cystitis episodes. Shortly after delivery, a common pathogen, *K. pneumoniae* was detected in this patient’s urine after sudden onset of acute cystitis. This pathogen was quickly eradicated with oral Cephalexin therapy, taken in form of monodose 1 g per day. Our study has shown that *K. pneumoniae* was extremely sensitive to this antibiotic since its relative abundance in urine plummeted from 94.1% to marginal 1.04% after just 2 days under therapy. However, *Lactobacillus* sp., although initially abundant were also depleted by the last day of therapy, making the urinary tract significantly more susceptible to reinfection. We believe that the significant decline in *Lactobacillus* sp. caused by 7-day therapy contributed to the development of *Candida* infection, which was the latter cause of recurring cystitis in this patient. Generalized Lotka-Volterra model based on QIIME microbiome analysis of raw DNA sequencing data collected from 28 patients predicted *Bacilli* class with *Lactobacillus* as dominant genus negatively impact on overgrowth of pathogens belonging to *Gammaproteobacteria* class. The second model based on longitudinal study data has further highlighted the role of *Gammaproteobacteria* class pathogens related to cystitis symptoms. However, this model is markedly different. This second model predicted *Gammaproteobacteria* class negative impact on the *Bacilli* class. Both models indicated members of *Actinobacteria* class as an important contributor to UT microbiota “equilibrium” both actively suppressing pathogen overgrowth and having a protective influence on *Lactobacillus* during treatment. The marked difference in reversed positive-negative interactions between *Bacilli* and *Gammaproteobacteria* classes in these two gLVM models could arguably be attributed to the introduction of antibiotic.

Overall, our results indicate this antibiotic was an excellent choice for rapid removal of pathogen from the patient’s UT, however, continuation of therapy after 3 days in this particular case has dramatically impacted host’s urinary microbiome, and enabled opportunistic pathogens from class *Pseudomonadaceae* and ultimately *C. albicans* to occupy their niches in UT and in the end cause cystitis symptoms to relapse. This strongly suggests that time under therapy is an equally important therapy parameter as the initial choice of antimicrobial drug. Our study suggests that introduction of genomics based methods, alongside traditional culture-based ones, can be a great aid in assessing this second parameter. We are fully aware that our study was limited in a sense that we monitored urinary microbiome change during antibiotic therapy in a single patient. In future, more patients with different therapy durations should be monitored and monitoring should be performed in prolonged periods after completing antibiotic therapy.

## Conclusions

In the present study, dysbiosis of UT microbiota associated with cystitis was characterized. Briefly, the results revealed increases in the abundances of bacteria associated with UT infections, decrease in the abundances of potential beneficial bacteria, and changes in the interactions of the constituent taxa making up microbiota in patients with cystitis. The two gLVM models based on 16S rDNA sequencing suggested that *Actinobacteria* phylum bacteria are interacting with both pathogen and beneficial representatives of UT microbiota. This highly diverse bacterial phylum characterized by extraordinary metabolic versatility and ability to produce most of the clinically used antibiotics and a plethora of other natural products is not sufficiently explored in regards to human UT and from this study it appears to harbor some highly beneficial representatives of healthy UT microbiota. Moreover, longitudinal microbiota insight obtained from a single patient under prescribed antimicrobial therapy revealed rapid and extensive changes in microbial composition of UT and emphasized the need for current guidelines revision in regards to Cephalexin therapy duration, although further studies are needed to explore this for other commonly prescribed antibiotics.

## Data Availability Statement

The datasets presented in this study can be found in online repositories. The names of the repository/repositories and accession number(s): https://www.ebi.ac.uk/, Project: PRJEB42164, deposited on 18-12-2020 (https://www.ebi.ac.uk/ena/browser/view/PRJEB42164).

## Ethics Statement

The studies involving urine samples from human participants were reviewed and approved by the Ethics Committee of University Hospital Dubrava affiliated with Faculty of Food Technology and Biotechnology and Faculty of Pharmacy and Biochemistry. The patients/participants provided their written informed consent to participate in this study.

## Author Contributions

AS and JZ performed the study design, data analysis, drafting, revising, final approval, and handled the accountability of all aspects of the work. EM, ES, and MC performed the bioinformatics analysis and data acquisition. MC, LS, and DO performed the data analysis and acquisition. AS, JZ, JS, KB, and MC performed the study design, clinical sample collection, data analysis, revising, and final approval and were also involved in the accountability of all aspects of the work. All authors contributed to the article and approved the submitted version.

## Funding

This study was supported by the Croatian Science Foundation under research project “Exploring Gut Microbiome Equilibrium” (Grant HRZZ_IP_06_2016_3509) and by the Croatian Government and the European Union through the European Regional Development Fund: Competitiveness and Cohesion Operational Programme (KK.01.1.1.01) The Scientific Centre of Excellence for Marine Bioprospecting–BioProCro.

## Conflict of Interest

The authors declare that the research was conducted in the absence of any commercial or financial relationships that could be construed as a potential conflict of interest.
