# Supplementary figures and images for: Modeling of Urinary Microbiota Associated With Cystitis
Source: Front Cell Infect Microbiol. 2021 Mar 16;11:643638. doi: 10.3389/fcimb.2021.643638 (PMC8008076; doi:10.3389/fcimb.2021.643638)

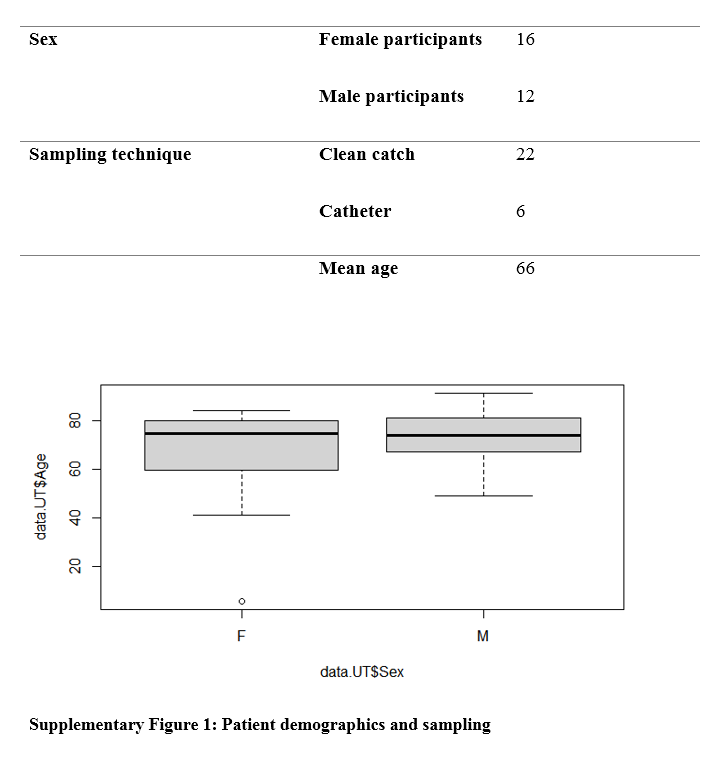

Supplement: Supplementary file 2 [file Image_1.tif]

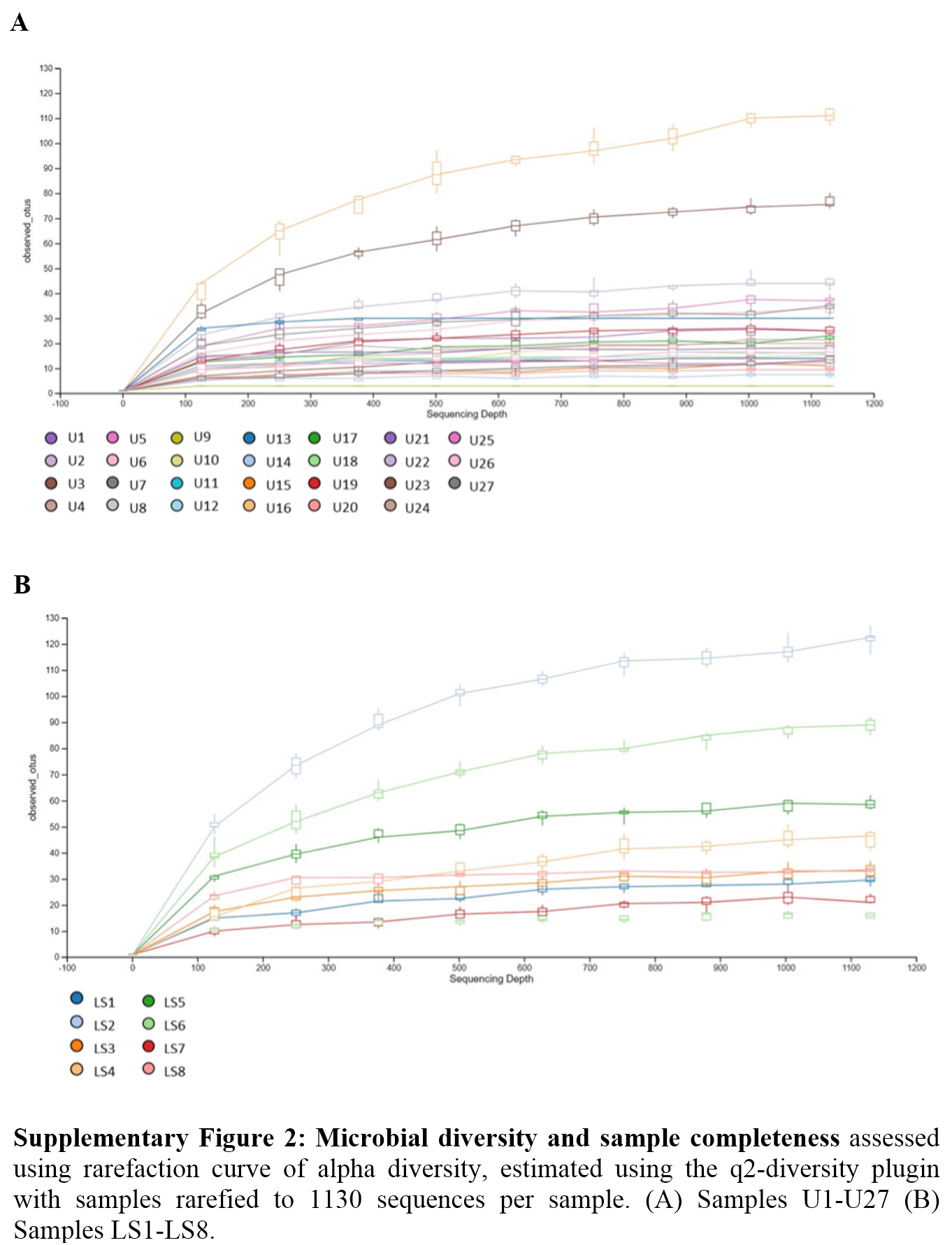

Supplement: Supplementary file 3 [file Image_2.tif]
