# Supplementary material for: Modeling of Urinary Microbiota Associated With Cystitis
Source: Front Cell Infect Microbiol. 2021 Mar 16;11:643638. doi: 10.3389/fcimb.2021.643638 (PMC8008076; doi:10.3389/fcimb.2021.643638)
Supplement: Supplementary file 4 [file Table_1.docx]

| **Number** | **Sample name** | **Urine culture test result** | **16S sequencing result** | **%** |
| --- | --- | --- | --- | --- |
| 1 | U1^#^ | *Acinetobacter baumannii (‎Moraxellaceae) , Streptoccocus species (Streptococcaceae)* | *Actinomycetaceae* | 18.51 |
|  |  |  | *Aerococcaceae* | 74.25 |
|  |  |  | *Lactobacillaceae* | 1.67 |
|  |  |  | *Veillonellaceae* | 2.61 |
|  |  |  | *Other* | 2.97 |
| 2 | U2 | *Proteus mirabilis (Enterobacteriaceae)* | *Porphyromonadaceae* | 4.06 |
|  |  |  | *Prevotellaceae* | 1.01 |
|  |  |  | *Tissierellaceae* | 15.01 |
|  |  |  | *Campylobacteraceae* | 3.33 |
|  |  |  | *Enterobacteriaceae* | 73.91 |
|  |  |  | *Other* | 2.69 |

| 3 | U3^#^ | *Citrobacter koseri (Enterobacteriaceae)* | *Actinomycetaceae* | 2.91 |  |
| --- | --- | --- | --- | --- | --- |
|  |  |  | *Porphyromonadaceae* | 1.78 |  |
|  |  |  | *Prevotellaceae* | 61.47 |  |
|  |  |  | *Peptococcaceae* | 1.16 |  |
|  |  |  | *Veillonellaceae* | 12.93 |  |
|  |  |  | *Tissierellaceae* | 6.93 |  |
|  |  |  | *Fusobacteriaceae* | 1.64 |  |
|  |  |  | *Campylobacteraceae* | 3.66 |  |
|  |  |  | *Moraxellaceae* | 1.27 |  |
|  |  |  | *Pseudomonadaceae* | 1.67 |  |
|  |  |  | *Other* | 4.59 |  |
| 4 | U4 | *Enterococcus faecalis (Enterococcaceae) , Escherichia coli (Enterobacteriaceae)* | *Enterococcaceae Other* | 97.20 2.8 |  |
|  |  |  |  |  |  |
| 5 | U5 | *Pseudomonas aeruginosa, (Pseudomonadaceae)* | *Bacteroidaceae* | 9.09 |  |
|  |  |  | *Porphyromonadaceae* | 1.79 |  |
|  |  |  | *Enterococcaceae* | 6.04 |  |
|  |  |  | *Tissierellaceae* | 2.34 |  |
|  |  |  | *Pseudomonadaceae* | 78.89 |  |
|  |  |  | *Other* | 1.85 |  |
| 6 | U6 | *Enterococcus faecium (Enterococcaceae), Morganella morganii (Enterobacteriaceae)* | *Enterobacteriaceae Enterococcaceae Other* | 81.66 15.29 3.04 |  |
|  |  |  |  |  |  |
| 7 | U7 | *Escherichia coli (Enterobacteriaceae), Klebsiella pneumoniae (Enterobacteriaceae)* |  |  |  |
|  |  |  | *Enterobacteriaceae Enterococcaceae Other* | 72.48 24.62 2.91 |  |
|  |  |  |  |  |  |
| 8 | U8 | *Pseudomonas aeruginosa, (Pseudomonadaceae)* | *Pseudomonadaceae* | 99.19 |  |
|  |  |  | *Other* | 0.81 |  |
| 9 | U9 | *Klebsiella pneumoniae (Enterobacteriaceae)* | *Enterobacteriaceae* | 100.00 |  |
| 10 | U10 | *Klebsiella oxytoca (Enterobacteriaceae)* | *Enterobacteriaceae* | 97.89 |  |
|  |  |  | *Other* | 2.11 |  |

| 11 | U11 | *Klebsiella pneumoniae ESBL (Enterobacteriaceae)* | *Carnobacteriaceae* | 4.35 |  |
| --- | --- | --- | --- | --- | --- |
|  |  |  | *Tissierellaceae* | 1.50 |  |
|  |  |  | *Enterobacteriaceae* | 91.80 |  |
|  |  |  | *Other* | 2.35 |  |
| 12 | U12 | *Proteus mirabilis (Enterobacteriaceae)* | *Enterobacteriaceae* | 99.32 |  |
|  |  |  | *Other* | 0.65 |  |
| 13 | U13^#^ | *Enterococcus faecalis (Enterococcaceae)* | *Cellulomonadaceae* | 1.56 |  |
|  |  |  | *Corynebacteriaceae* | 3.04 |  |
|  |  |  | *Microbacteriaceae* | 1.22 |  |
|  |  |  | *Propionibacteriaceae* | 13.45 |  |
|  |  |  | *Cytophagaceae* | 7.99 |  |
|  |  |  | *Staphylococcaceae* | 6.16 |  |
|  |  |  | *Aerococcaceae* | 2.34 |  |
|  |  |  | *Lactobacillaceae* | 11.89 |  |
|  |  |  | *Clostridiales** | 2.08 |  |
|  |  |  | *Lachnospiraceae* | 2.26 |  |
|  |  |  | *Peptostreptococcaceae* | 1.04 |  |
|  |  |  | *Erysipelotrichaceae* | 3.21 |  |
|  |  |  | *Sphingomonadaceae* | 1.56 |  |
|  |  |  | *Alcaligenaceae* | 1.22 |  |
|  |  |  | *Comamonadaceae* | 6.51 |  |
|  |  |  | *Moraxellaceae* | 8.33 |  |
|  |  |  | *Pseudomonadaceae* | 20.05 |  |
|  |  |  | *Xanthomonadaceae* | 4.60 |  |
|  |  |  | *Other* | 1.48 |  |
| 14 | U14 | *Enterococcus faecalis (Enterococcaceae)* | *Enterococcaceae* | 99.65 |  |
|  |  |  | *Other* | 0.35 |  |
| 15 | U15 | *Enterobacter cloaceae (Enterobacteriaceae)* | *Enterobacteriaceae* | 99.24 |  |
|  |  |  | *Other* | 0.76 |  |
| 16 | U16 | *Citrobacter koseri (Enterobacteriaceae)* | *Bacteroidales** | 1.97 |  |
|  |  |  | *Bacteroidaceae* | 3.66 |  |
|  |  |  | *Porphyromonadaceae* | 8.01 |  |
|  |  |  | *Rikenellaceae* | 2.40 |  |
|  |  |  | *Christensenellaceae* | 1.71 |  |
|  |  |  | *Lachnospiraceae* | 2.86 |  |
|  |  |  | *Ruminococcaceae* | 2.65 |  |
|  |  |  | *Erysipelotrichaceae* | 1.03 |  |
|  |  |  | *Desulfovibrionaceae* | 1.62 |  |
|  |  |  | *Enterobacteriaceae* | 67.04 |  |
|  |  |  | *Other* | 7.06 |  |

| 17 | U17 | *Proteus mirabilis (Enterobacteriaceae)* | *Enterobacteriaceae* | 98.24 |  |
| --- | --- | --- | --- | --- | --- |
|  |  |  | *Other* | 1.76 |  |
| 18 | U18 | *Proteus mirabilis (Enterobacteriaceae)* | *Enterobacteriaceae* | 99.16 |  |
|  |  |  | *Other* | 0.84 |  |
| 19 | U19 | *Escherichia coli (Enterobacteriaceae), Proteus mirabilis (Enterobacteriaceae)* | *Enterobacteriaceae Other* | 97.02 2.98 |  |
|  |  |  |  |  |  |
| 20 | U20 | *Proteus mirabilis Enterobacteriaceae* | *Enterobacteriaceae* | 99.65 |  |
|  |  |  | *Other* | 0.35 |  |
| 21 | U21 | *Enterobacter aerogenes (Enterobacteriaceae)* | *Lactobacillaceae* | 19.40 |  |
|  |  |  | *Enterobacteriaceae* | 80.03 |  |
|  |  |  | *Other* | 0.57 |  |
| 22 | U22 | *Escherichia coli (Enterobacteriaceae)* | *Enterobacteriaceae* | 99.25 |  |
|  |  |  | *Other* | 0.75 |  |
| 23 | U23 | *Enterobacter cloacae (Enterobacteriaceae)* | *Enterobacteriaceae* | 95.99 |  |
|  |  |  | *Other* | 4.01 |  |
| 24 | U24 | *Enterobacter cloacae (Enterobacteriaceae); Enterococcus faecalis (Enterococcaceae), Escherichia coli (Enterobacteriaceae), Proteus mirabilis (Enterobacteriaceae)* | *Enterococcaceae Enterobacteriaceae Other* | 1.02 96.92 3.08 |  |
|  |  |  |  |  |  |
| 25 | U25 | *Escherichia coli (Enterobacteriaceae), Klebsiella pneumoniae (Enterobacteriaceae)* | *Enterobacteriaceae Other* | 99.72 0.28 |  |
|  |  |  |  |  |  |
| 26 | U26 | *Escherichia coli (Enterobacteriaceae)* | *Enterobacteriaceae* | 97.34 |  |
|  |  |  | *Other* | 2.66 |  |
| 27 | U27 | *Escherichia coli (Enterobacteriaceae)* | *Enterobacteriaceae* | 99.19 |  |
|  |  |  | *Other* | 0.81 |  |
| 28 | LS1 | *Klebsiella pneumoniae (Enterobacteriaceae)* | *Enterobacteriaceae Pseudomonadaceae* Other | 95.41 1.31 3.28 |  |

*# Bacterial family identified by 16S rRNA sequening was not in accordance with standard urine culture test*

**Represents Taxonomic order, family was not assigned by 16S rRNA sequening*

**Supplementary Table 1**: Comparison of results obtained by standard urine culture tests and 16S rRNA sequencing. The 16S rRNA sequencing column depicts most abundant taxa on family level.
